# Angiotensin‐(1–9) Improves the Cardioprotective Effects of Del Nido Cardioplegia Against Ischemia/Reperfusion Injury

**DOI:** 10.1111/jcmm.70412

**Published:** 2025-02-12

**Authors:** Evelyn Mendoza‐Torres, Gina Sanchez, Wendy Rosales, María Clara Ospino, Luis Antonio Díaz‐Ariza, Yuliet Montoya, John Bustamante, Jaime A. Riquelme, Mario Chiong, Sergio Lavandero

**Affiliations:** ^1^ Grupo de Investigación Avanzada en Biomedicina, Faculty of Health, Exact and Natural Sciences Universidad Libre Seccional Barranquilla Barranquilla Colombia; ^2^ Programa de Fisiopatología, Instituto de Ciencias Biomédicas, Facultad de Medicina Universidad de Chile Santiago Chile; ^3^ Internal Medicine Program, Faculty of Health Sciences Universidad Libre Seccional Barranquilla Barranquilla Colombia; ^4^ Grupo de Dinámica Cardiovascular, Centro de Bioingeniería Universidad Pontificia Bolivariana Medellín Colombia; ^5^ Laboratory of Cardiovascular Pharmacotherapy, Departamento de Química Farmacológica y Toxicológica, Facultad de Ciencias Químicas y Farmacéuticas Universidad de Chile Santiago Chile; ^6^ Interuniversity Center for Healthy Aging Santiago Chile; ^7^ Advanced Center for Chronic Diseases (ACCDiS), Facultad de Ciencias Químicas y Farmacéuticas & Facultad de Medicina Universidad de Chile Santiago Chile; ^8^ Department of Internal Medicine (Cardiology Division) University of Texas Southwestern Medical Center Dallas Texas USA

**Keywords:** angiotensin‐(1–7), angiotensin‐(1–9), del Nido cardioplegia, heart, ischemia/reperfusion, myocardial function, renin‐angiotensin system

## Abstract

Del Nido cardioplegia (DNC), a blood‐and‐crystalloid solution containing high and low concentrations of potassium and calcium, respectively, is used as a single‐dose antegrade infusion to induce immediate cardiac arrest in the surgery of patients with cardiovascular diseases requiring extracorporeal circulation. Adding cardioprotective molecules may further reduce the damage‐triggered ischemia/reperfusion (I/R) injury. Angiotensin‐(1–9) (Ang‐(1–9)) and angiotensin‐(1–7) (Ang‐(1–7)), members of the counter‐regulatory renin‐angiotensin system, have shown cardioprotective effects in cardiac hypertrophy and I/R models. This study aimed to evaluate the effects of Ang‐(1–9) and Ang‐(1–7), as adjuvants of the DNC, on cardioprotection and ventricular function in isolated rat hearts subjected to I/R and in cultured neonatal rat ventricular myocytes subjected to simulated I/R (sI/R). The addition of DNC and Ang‐(1–9) and Ang‐(1–7) decreased lactic dehydrogenase (LDH) release in cultured cardiomyocytes subjected to sI/R in comparison to those cardiomyocytes subjected to sI/R and incubated with DNC alone. Moreover, hearts treated with Ang‐(1–9) during reperfusion after DNC + I/R exhibited fewer arrhythmias and required less time to reach left ventricular developed pressure stability. Overall, reperfusion with DNC and Ang‐(1–9) improves the recovery of the left ventricular function of the heart.

## 1 Introduction

Cardiac surgery requires extracorporeal circulation to provide cardiopulmonary assistance to the patient [1]. However, this procedure implies exposing the myocardium to ischemia/reperfusion (I/R) injury [2]. Cardioplegia, a standard method of cardioprotection, consists of cardiac arrest using a hyperkalemic solution [2, 3].

Del Nido cardioplegia (DNC) is used for myocardial protection during cardiac surgery and results in lower troponin release in paediatric patients compared to standard cardioplegia [4, 5]. However, this cardioplegia faces the challenge of conferring greater cardioprotection in elderly patients or patients with left ventricular hypertrophy secondary to hypertension or myocardial infarction [6]. Thus, there is a current need to improve the protective effects of DNC.

Angiotensin‐(1–9) [Ang‐(1–9)] and angiotensin‐(1–7) [Ang‐(1–7)] are vasoactive peptides of the counter‐regulatory renin‐angiotensin system with known cardioprotective effects in murine models [7, 8]. Ang‐(1–9) reduces cardiac dysfunction in diabetic rats and attenuates hypertension in rats by improving cardiac and endothelial function [9, 10] and Ang‐(1–7) increases insulin sensitivity and decreases systolic blood pressure [8]. Therefore, we evaluated whether Ang‐(1–9) or Ang‐(1–7) can enhance the cardioprotective effects of DNC using in vitro and ex vivo models of I/R injury.

## 2 Methods

This study conformed to the Guide for the Care and Use of Laboratory Animals, published by the U.S. National Institutes of Health (NIH, Publication No. 85–23, revised in 1996), and was approved by the Institutional Ethics Review Committee from the Faculty of Medicine of Universidad de Chile (Protocol number CBA 22547‐MED‐UCH) and the Institutional Ethics Review Committee from Universidad Libre‐Barranquilla, Colombia (Ethics Number: 0041–2019).

DNC was prepared according to Govindapillai et al. 2013 [4]. The base solution was Plasma‐Lyte A (Baxter Healthcare Corporation, USA) with the following additives: KCl 2 mEq/mL, NaHCO_3_ 1 mEq/mL, MgSO_4_ 0.2 g/mL, lidocaine 1%, mannitol 25%.

Neonatal rat ventricular myocytes (NRVM) were isolated from one‐to three‐day‐old Sprague Dawley rats (Appendix). The sI/R protocol was applied according to Mendoza‐Torres et al. (2018) [7]. Ischemia was induced by incubating the cardiomyocytes in an ischemia‐mimicking solution under O_2_ < 1%, 5% CO_2_ and 95% Nitrogen at 37°C for 8 h. Subsequently, for simulated reperfusion, the ischemia‐mimicking solution was replaced by DMEM/M199 (4:1) containing 10% FBS and NRVM were incubated for 16 h in 95% air and 5% CO_2_. Parallel NRVM were assigned to a control group exposed to normoxic conditions under 95% air and 5% CO_2_ for 8 h followed by simulated reperfusion. The experimental conditions are presented in the Appendix.

Lactate dehydrogenase (LDH) activity was measured in the culture medium after normoxia and sI/R using the CytoTox 96 Non‐Radioactive Cytotoxicity Assay, Promega (Madison, WI, USA). Apoptosis was evaluated with the in situ Cell Death Detection Kit, Fluorescein (Roche, Penzberg, Germany). Images were captured with ZOE Fluorescent Cell Imager (Bio‐Rad).

Adult male Sprague–Dawley rats (250–300 g) were anaesthetised with pentobarbital [80 mg/kg intraperitoneally (i.p.)], and heparin 100 U/kg was injected into the right atria. Hearts were harvested and perfused through the aorta with Krebs–Henseleit (KH) solution (equilibrated with a gas mixture of 95% O_2_ and 5% CO_2_ at 37°C), using a peristaltic pump (Gilson Miniplus 3, France) according to Mendoza‐Torres et al. (2018) [7]. Hearts were subjected to 30 min of global ischemia followed by 60 min of reperfusion. Left ventricular developed pressure (LVDP) and left ventricular end‐diastolic pressure (LVEDP) were monitored continuously with pressure transducers.

The results are presented as mean ± SEM. Non‐parametric statistical analysis for multiple comparisons and Tukey's post‐test were performed with GraphPad Prism for Windows Version 10.1.1 (GraphPad Software Inc., La Jolla, CA, USA).

## 3 Results

### 3.1 Ang‐(1–9) and Ang‐(1–7) With DNC Improves Cardiomyocyte Survival During I/R

**FIGURE 1:**
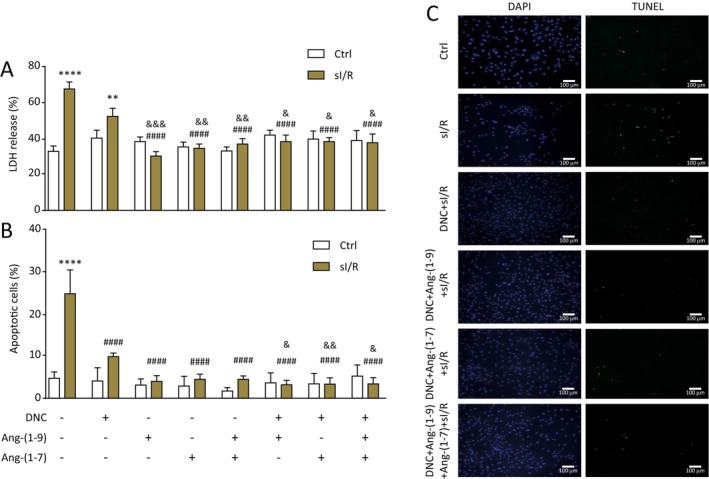
Adding Ang‐(1–9) and Ang‐(1–7) to DNC decreases cell death in neonatal rat ventricular cardiomyocytes (NRVM) subjected to sI/R. NRVM were subjected to sI/R (ischemia by 8 h and reperfusion by 16 h in the absence or presence of different treatments: Ang‐(1–9) 1 μM, Ang‐(1–7) 10 nM, Ang‐(1–9) + Ang‐(1–7), DNC, DNC + Ang‐(1–9), DNC + Ang‐(1–7) and DNC + Ang‐(1–9) + Ang‐(1–7). (A) LDH release was determined as an index of cell death and was expressed as a % of total LDH activity. (B,C) Percentage of TUNEL‐positive nuclei was determined by immunofluorescence. Absolute values of 5–10 separate fields were averaged, and apoptotic cells were expressed as a percentage of total cells in five independent experiments. The mean viability of the primary culture without treatment was 4%, according to the percentage of TUNEL‐positive nuclei. Data expressed as mean ± SEM (*n* = 4 independent experiments). ANOVA and Tukey's post‐test were applied. **p < 0.01 versus control without treatment, ****p < 0.0001 versus control without treatment, ^####^ *p* < 0.0001 versus I/Rs without treatment, ^&^ *p* < 0.05 versus DNC + sI/R, ^&&^ *p* < 0.01 versus DNC + sI/R, ^&&&^ *p* < 0.001 versus DNC + sI/R. DNC, Del Nido cardioplegia; LDH, Lactate dehydrogenase; sI/R: Simulated ischemia/reperfusion.

NRVM were subjected to ischemia in the presence or absence of DNC with or without Ang‐(1–9) 1 μM or Ang‐(1–7) 100 nM or the combination of these peptides for 8 h followed by 16 h of reperfusion with or without Ang‐(1–9) or Ang‐(1–7) or the two peptides together and LDH release was determined. Figure 1A shows that cardiomyocytes subjected to sI/R without treatment presented a higher percentage of LDH release than the control condition (*p* < 0.05). The cardiomyocytes subjected to DNC + sI/Rs did not show a significant statistical difference from those subjected to sI/R. However, NRVM incubated with DNC plus any of the two peptides or the combination of these showed less LDH release (*p* < 0.05) (Figure 1A). Furthermore, we observed a lower percentage of TUNEL‐positive cells in cardiomyocytes subjected to sI/R + DNC + Ang‐(1–9) or Ang‐(1–7) in comparison with cardiomyocytes subjected to sI/R without treatment (Figure 1B,C).

### 3.2 Ang‐(1–9) and Ang‐(1–7) Improve Recovery of the Left Ventricular Function in Isolated Rat Hearts Treated With DNC and Subjected to I/R

**FIGURE 2:**
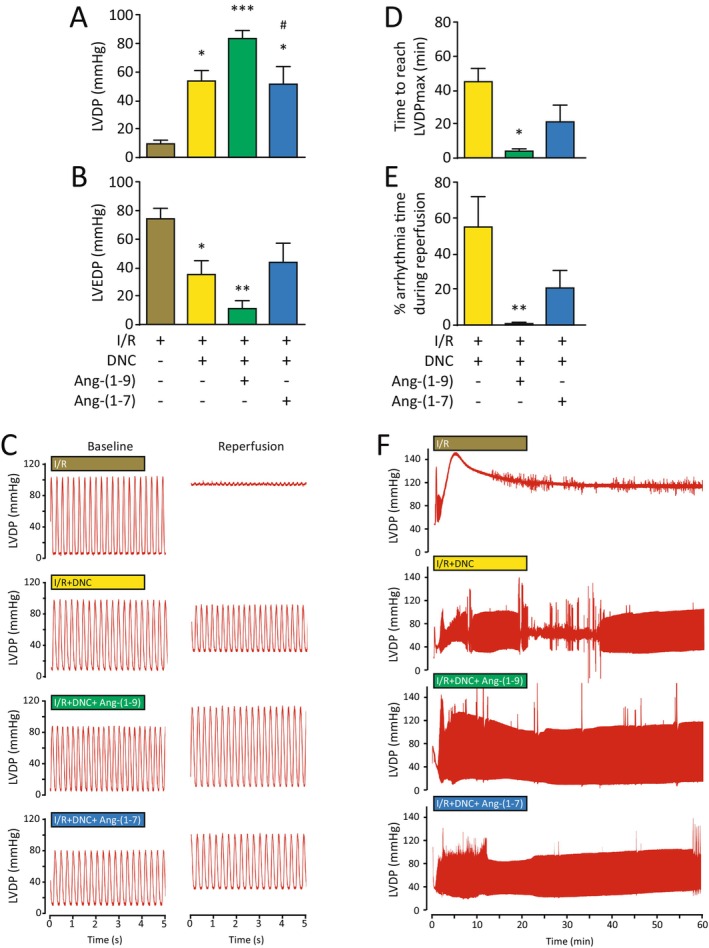
Ang‐(1–9) improves left ventricular function and decreases the recovery time and arrhythmias in isolated rat hearts subjected to DNC + I/R. (A) LVDP and (B) LVEDP measured at the end of reperfusion *p < 0.05 versus I/R, **p < 0.01 versus I/R, ***p < 0.001 versus I/R, ^#^ *p* < 0.05 versus DNC + Ang‐(1–9); (C) Representative tracings of LVDP recovery following reperfusion to baseline; (D) Time to reach the LVDPmax, (E) Percentage of arrhythmia time during reperfusion *p < 0.05 versus DNC + I/R, **p < 0.01 versus DNC + I/R and (F) Representative tracings of LVDP during 1 h reperfusion. Bar graphs represent mean ± SEM. N=5 hearts in each condition. Data were analysed by ANOVA followed by Tukey's post‐test. I/R, Ischemia/reperfusion; LV, Left ventricular; LVDP, LV developed pressure and LVEDP, LV end‐diastolic pressure.

We tested the potential additive effects of these peptides on DNC in an ex vivo model of I/R injury. Treatment with DNC, DNC + Ang‐(1–9) or DNC + Ang‐(1–7) improved the LVDP during reperfusion in isolated rat hearts subjected to global I/R (Figure 2A,C). However, there was no significant difference between these treatments (*p* > 0.05). Moreover, hearts treated with DNC or DNC + Ang‐(1–9) showed a decrease in the LVEDP compared to those subjected to I/R (I/R vs. DNC *p* < 0.05 and I/R vs. DNC + Ang‐(1–9) + I/R [*p* < 0.001]) (Figure 2B,C). However, there was no difference between both treatments (*p* > 0.05). In addition, treatment with DNC + Ang‐(1–7) was not as effective as treatment with DNC + Ang‐(1–9) in the improvement of LVDP and hearts subjected to I/R (*p* < 0.05) (Figure 2A–C). Treatment with DNC + Ang‐(1–9) followed by I/R required less time to reach pressure stability and exhibited fewer arrhythmias during reperfusion in comparison with isolated rat hearts treated with DNC and subjected to I/R (*p* < 0.01) (Figure 2D–F).

## 4 Discussion

Ang‐(1–9) and Ang‐(1–7) improved the protective effect of DNC during sI/R. Indeed, DNC alone reduces cell death, but this reduction is increased with these peptides. Therefore, these peptides may be used as protective strategies in cardiac arrest.

A meta‐analysis with 21.779 patients demonstrated that DNC reduced short‐term adverse outcomes by reducing in post‐surgical cardiac enzymes, aortic clamping time and cardioplegia volume [11]. In this study, DNC showed improvement of LVDP and LEVDP in isolated rat hearts subjected to ischemia, as has been previously described [12]. Our results demonstrate that DNC ensures recovery of the LVDP in hearts subjected to ischemia and that it can also be improved with the use of Ang‐(1–9), leading to a decrease in arrhythmias and quick recovery of left ventricular function during reperfusion.

Cardiac arrhythmias represent one of the most common complications after open heart surgery and are an essential factor in mortality and morbidity [13]. Unlike Ang‐(1–7) and Ang‐(1–9) reduced the incidence of arrhythmias and promoted a quick recovery of the initial LVDP, which may be due to the different signalling pathways activated by each peptide [7, 14], the specifical mechanisms mediating these protective effects remain to be elucidated. Fattah et al. (2016) showed that Ang‐(1–9) preserved left ventricular systolic function in a murine model of myocardial infarction and exerted a positive inotropic effect in adult mice cardiomyocytes by increasing calcium transient amplitude and contractility but whether the regulation of calcium handling can account for the peptide's anti‐arrhythmogenic effect should be addressed by future research [15].

An important limitation of our work is the lack of an in vivo assessment of the combination of these peptides and DNC, but the protective effect of both Ang‐(1–7) and Ang‐(1–9) in vivo have been well‐established [7, 9, 10, 15]. Our preliminary data suggests an additive effect of Ang‐(1–9) and DNC, but future studies should thoroughly confirm these findings.

Overall, Ang‐(1–9) may be considered a potential adjuvant agent to enhance DNC by improving left ventricular function and reducing the incidence of postoperative ventricular arrhythmias, thereby minimising the use of extracorporeal circulation devices, reducing the length of Intensive Care Unit stay and improving the overall outcome of the patients undergoing cardiovascular surgery.

## Author Contributions

**Evelyn Mendoza‐Torres:** conceptualization (lead), formal analysis (lead), funding acquisition (lead), investigation (lead), methodology (lead), project administration (lead), resources (lead), software (equal), supervision (equal), validation (equal), visualization (equal), writing – original draft (lead), writing – review and editing (lead). **Gina Sanchez:** formal analysis (equal), investigation (equal), methodology (equal), software (equal), writing – review and editing (equal). **Wendy Rosales:** conceptualization (equal), funding acquisition (equal), methodology (equal), project administration (equal), supervision (equal), writing – review and editing (equal). **María Clara Ospino:** investigation (equal), visualization (equal), writing – original draft (equal), writing – review and editing (equal). **Luis Antonio Díaz‐Ariza:** investigation (equal), visualization (equal), writing – original draft (equal), writing – review and editing (equal). **Yuliet Montoya:** investigation (equal), methodology (equal), writing – original draft (equal), writing – review and editing (equal). **John Bustamante:** conceptualization (equal), investigation (equal), methodology (equal), writing – original draft (equal), writing – review and editing (equal). **Jaime A. Riquelme:** conceptualization (equal), methodology (equal), visualization (equal), writing – original draft (equal), writing – review and editing (equal). **Mario Chiong**: review and editing (equal). **Sergio Lavandero:** conceptualization (equal), investigation (equal), methodology (equal), writing – original draft (equal), writing – review and editing (equal).

## Ethics Statement

This study was approved by the Bioethics Committee from Universidad Libre‐Barranquilla, Colombia.

## Conflicts of Interest

The authors declare no conflicts of interest.

## Data Availability

The data that support the findings of this study are available upon request.
